# The Relationship Between Brain Frailty and Physical Function in Patients With Stroke Undergoing Rehabilitation

**DOI:** 10.7759/cureus.80453

**Published:** 2025-03-12

**Authors:** Motoki Maruyama, Sota Kajiwara, Takuto Oikawa, Masahiro Sasaki

**Affiliations:** 1 Rehabilitation, Akita Cerebrospinal and Cardiovascular Center, Akita, JPN; 2 Rehabilitation Medicine, Akita Cerebrospinal and Cardiovascular Center, Akita, JPN

**Keywords:** activities of daily living, brain frailty, physical function, rehabilitation, stroke

## Abstract

Background

Brain frailty has gained attention as a predictor of poor functional outcomes. However, the relationship between brain frailty and physical function among patients with stroke undergoing rehabilitation remains unclear. This study aimed to investigate the relationship between brain frailty and activities of daily living (ADLs) at discharge among patients with stroke admitted to a convalescent rehabilitation ward.

Methods

This single-center retrospective cohort study included patients with stroke admitted to the convalescent rehabilitation ward. Brain frailty (i.e., white matter hyperintensity, old vascular lesions, and brain atrophy) was evaluated using cranial magnetic resonance imaging at stroke onset. The outcome measure was defined as ADLs at discharge, assessed using the motor item of the Functional Independence Measure (FIM-M). Multiple regression and mediation analyses were performed to assess the association between brain frailty scores and FIM-M scores at discharge.

Results

The final analysis included 160 patients (median age: 73.0 years; interquartile range: 64.0-80.0 years; male: n = 90, 56.2%). The multiple regression analysis revealed that severe brain frailty (score of 3) was significantly associated with FIM-M scores at discharge, even after adjusting for covariates (β = −0.18; p = 0.041). Furthermore, mediation analysis revealed that severe brain frailty was associated with FIM-M scores at discharge through the mediation of cognitive function (total effect = −16.20; p < 0.001).

Conclusions

Brain frailty may provide new insights for outcome prediction in stroke rehabilitation, highlighting the importance of incorporating its assessment into routine clinical practice.

## Introduction

Frailty has become a critical issue in the field of rehabilitation. This condition is characterized by a multifaceted biopsychosocial syndrome encompassing physical, cognitive, and social vulnerabilities, indicating a pre-disability condition [1]. The prevalence of physical frailty has been reported to be 17.4% among older adults [1], increasing to 27.0% among patients with stroke [2]. Furthermore, frailty is closely associated with other age-related conditions, such as sarcopenia, undernutrition, and low physical activity levels, contributing to a vicious cycle [3]. In patients with stroke, who often have multiple comorbidities associated with aging, frailty has been increasingly recognized as a significant therapeutic target. Previous studies have reported that frailty in patients with stroke is associated with disease severity [4], mortality [5], impaired activities of daily living (ADLs) [6], lower quality of life (QOL) [7], and poorer functional outcomes [8].

Recently, the concept of brain frailty, in addition to physical, cognitive, and social frailty, has gained attention as an emerging condition [9,10]. Brain frailty comprises imaging markers obtained from cranial magnetic resonance imaging (MRI) or computed tomography (CT) scans, including old vascular lesions (e.g., lacunes, old infarcts, and cerebral microbleeds), white matter hyperintensities (WMH), brain atrophy, and enlarged perivascular spaces [9-14]. Patients with stroke often undergo cranial MRI or CT scans early in the course of the disease for diagnostic and differential diagnostic purposes, allowing for the assessment of brain frailty without the need for further evaluation. Previous studies have reported that brain frailty is associated with physical frailty [12], cognitive impairment [9], stroke events [13], and the modified Rankin scale (mRS) score 90 days after stroke onset [9,11]. Furthermore, brain frailty has been reported to be a mediating factor between age and functional outcomes, and it holds potential as a prognostic indicator in aging populations [15]. Based on previous research findings, brain frailty may negatively affect rehabilitation outcomes in patients with stroke who require long-term interventions. Furthermore, as brain frailty adversely affects cognitive function [9], it is necessary to investigate the potential mediating role of cognitive function in the relationship between brain frailty and physical function. However, the specific effects of brain frailty on outcomes in patients with stroke undergoing rehabilitation remain unclear. Brain frailty, which is an easily obtainable measure in routine clinical practice, may serve as a valuable adjunctive marker for predicting rehabilitation outcomes. Furthermore, brain frailty is a common condition among patients with stroke and has the advantage of allowing objective assessment based solely on brain MRI images, without the need for questionnaire-based indices or performance evaluations, as required in conventional frailty assessments. Investigating the relationship between brain frailty, physical function, and cognitive function may provide insights into a comprehensive understanding of how brain frailty affects rehabilitation outcomes.

This study aimed to investigate the association between brain frailty, assessed using MRI at stroke onset, and physical function at discharge in patients with stroke admitted to a convalescent rehabilitation ward. The hypothesis of this study posits that severe brain frailty is associated with ADLs at discharge and that cognitive function partially mediates this association.

## Materials and methods

Study design and subject

This single-center retrospective cohort study was conducted at an institution with acute and convalescent rehabilitation wards in Japan. This study included patients admitted to the convalescent rehabilitation ward from the acute care ward of our hospital between July 2021 and June 2024. The inclusion criteria were as follows: patients with stroke aged 18 years or older. The exclusion criteria were as follows: (1) absence of cranial MRI within 24 hours of onset or insufficient imaging data; (2) admission to the convalescent rehabilitation ward ≥1 month after stroke onset, which may be influenced by prolonged hospitalization due to acute-phase complications or delays in transfer to the convalescent rehabilitation ward; (3) discharge within 30 days of admission to the convalescent rehabilitation ward or transfer to an acute hospital to treat other diseases, which may be affected by factors such as the selection of discharge destinations, waiting periods for transfer to care facilities, or the need to discontinue rehabilitation due to the treatment of acute-phase conditions; (4) premorbid ADL dependency (mRS ≥3); (5) presence of other neurological diseases; (6) subarachnoid hemorrhage; and (7) missing data. This study included patients with moderate to severe cognitive impairment. This study was conducted according to the Strengthening the Reporting of Observational Studies in Epidemiology guidelines.

Data collection

Data collection was conducted from July 2024 to September 2024. Demographic and clinical data, including age, sex, body mass index (BMI), stroke subtype, days from onset of the diseases to admission to the convalescent rehabilitation ward, premorbid ADL, history of stroke, comorbidities, lower limb motor paralysis, and ADL at admission to the convalescent rehabilitation ward, were collected from electronic medical records. The severity of motor paralysis was assessed using the Brunnstrom Recovery Stage (BRS) [16]. The premorbid status and admission ADLs were evaluated using the mRS and Functional Independence Measure (FIM), respectively [17,18]. Comorbidities were assessed using the Charlson Comorbidity Index (CCI) [19]. All demographic and clinical data were evaluated at the time of admission to the convalescent rehabilitation ward.

Brain frailty assessment

Brain frailty was assessed using a head MRI obtained within 24 h of stroke onset using a 1.5- or 3-Tesla MRI. The brain frailty was evaluated by a physical therapist (M.M.) and a stroke specialist with ≥20 years of experience (M.S.), based on radiologists’ reports, and the final diagnosis was made by a physician. The inter-rater reliability ICC (2,1) for the evaluation of brain frailty was 0.93 (95% CI: 0.90-0.95). In cases where discrepancies arose among evaluators, the physician's diagnosis was adopted. Brain frailty was defined based on three components: WMH, old vascular lesions (i.e., lacunes, old infarcts, and cerebral microbleeds), and brain atrophy [13]. WMH was assessed using T2-weighted or fluid-attenuated inversion recovery (FLAIR) images. Both periventricular WMH (PWMH) and deep WMH (DWMH) were evaluated using the Fazekas grading scale [20]. WMH presence was defined as follows: 0, no lucency; 1, lucency restricted to regions adjacent to the ventricles or extending throughout the entire region from the lateral ventricles to the cortex (Fazekas grade ≥1) [13]. Old vascular lesions were assessed using T2-weighted, FLAIR, and T2*-weighted images. Lacunes and old infarcts were identified as high signals on T2-weighted images or low signals on FLAIR images [21]. Cerebral microbleeds were detected as low signals on T2*-weighted images [21]. Old vascular lesions were classified as follows: 0, no old vascular lesion; 1, presence of either lacunes, old infarcts, or cerebral microbleeds in any location [13]. Brain atrophy was evaluated in both the cortical and central regions. The presence of brain atrophy was defined as follows: 0, absent; 1, presence of brain atrophy in either region [13]. The total brain frailty score was determined by assigning 1 point for each component: WMH, old vascular lesions, and brain atrophy [13]. The scores ranged from 0 to 3 points [13].

Outcomes

The outcome measure was defined as ADLs at discharge from the convalescent rehabilitation ward, assessed using the motor domain of the FIM (FIM-M) [18]. The FIM is a widely used tool for evaluating ADLs in patients with stroke. It comprises 13 items in the motor domain and five items in the cognitive domain (FIM-C), each scored on a seven-point scale [18]. The FIM-M and FIM-C have score ranges of 13-91 and 5-35 points, respectively, with total scores ranging from 18 to 126 points. In this study, FIM-M was used as an indicator of physical function, and FIM-C was used as an indicator of cognitive function. The FIM-M includes several components, such as self-care, sphincter control, transfers, and locomotion [18]. The FIM was assessed by experienced ward nurses within one week after admission to the convalescent rehabilitation ward and within one week before discharge.

Rehabilitation program

During hospitalization, the participants received up to three hours of rehabilitation interventions per day, including physical therapy, occupational therapy, and, when necessary, speech and language therapy. These rehabilitation programs were conducted through one-on-one sessions. For example, physical therapy involved range-of-motion exercises, stretching, resistance training, gait training, aerobic exercises, and ADL training.

Sample size calculation

The sample size was determined using G*Power (version 3.1; Heinrich-Heine-Universität Düsseldorf, Düsseldorf, Germany). For the calculations, an alpha error of 0.05, a power of 0.80, and an effect size (f²) of 0.15 were specified. With the number of independent variables set to 14 for the multiple regression analysis, the required total sample size to achieve sufficient power for rejecting the null hypothesis was 135 participants.

Statistical analysis

Statistical analyses were performed using IBM SPSS Statistics for Windows, Version 28 (Released 2021; IBM Corp., Armonk, New York). Parametric variables are presented as means and standard deviations, whereas nonparametric variables are presented as medians and interquartile ranges (IQRs). Categorical variables are presented as numbers and percentages. To compare baseline characteristics and outcomes between different brain frailty scores, the Kruskal-Wallis test, one-way analysis of variance, chi-square test, and Fisher’s exact test were performed. Multiple regression analysis was conducted to assess the association between brain frailty and FIM-M scores at discharge. The dependent variable was the FIM-M score, whereas the independent variables included brain frailty scores, using a score of 0 as the reference. The analysis was adjusted for potential confounders, including age, sex, stroke subtype, days from onset, history of stroke, premorbid mRS, CCI, lower limb BRS, and FIM-M and FIM-C scores at admission [22-25]. Multicollinearity was evaluated using the variance inflation factor (VIF), with a VIF <10 indicating no multicollinearity. A previous study reported that brain frailty was associated with cognitive function [9]. Thus, this study investigated the mediating effect of cognitive function (FIM-C) on the relationship between brain frailty and ADLs at discharge using mediation analysis. The PROCESS macro for SPSS (version 4.2) was employed for the analysis [26]. To test the significance of the indirect effect, a bootstrapping procedure with 5,000 resamples was performed. The mediation effect was considered statistically significant if the 95% confidence interval (CI) did not include zero. The significance level was set at 0.05.

Ethics

This study provided written information to the participants, clearly informing them of their right to withdraw at any time (opt-out) without any disadvantages. Personal information was handled with strict confidentiality according to the Declaration of Helsinki. The study was approved by the Ethics Committee of the Akita Cerebrospinal and Cardiovascular Center (Approval Number: 24-26).

## Results

**Figure 1:**
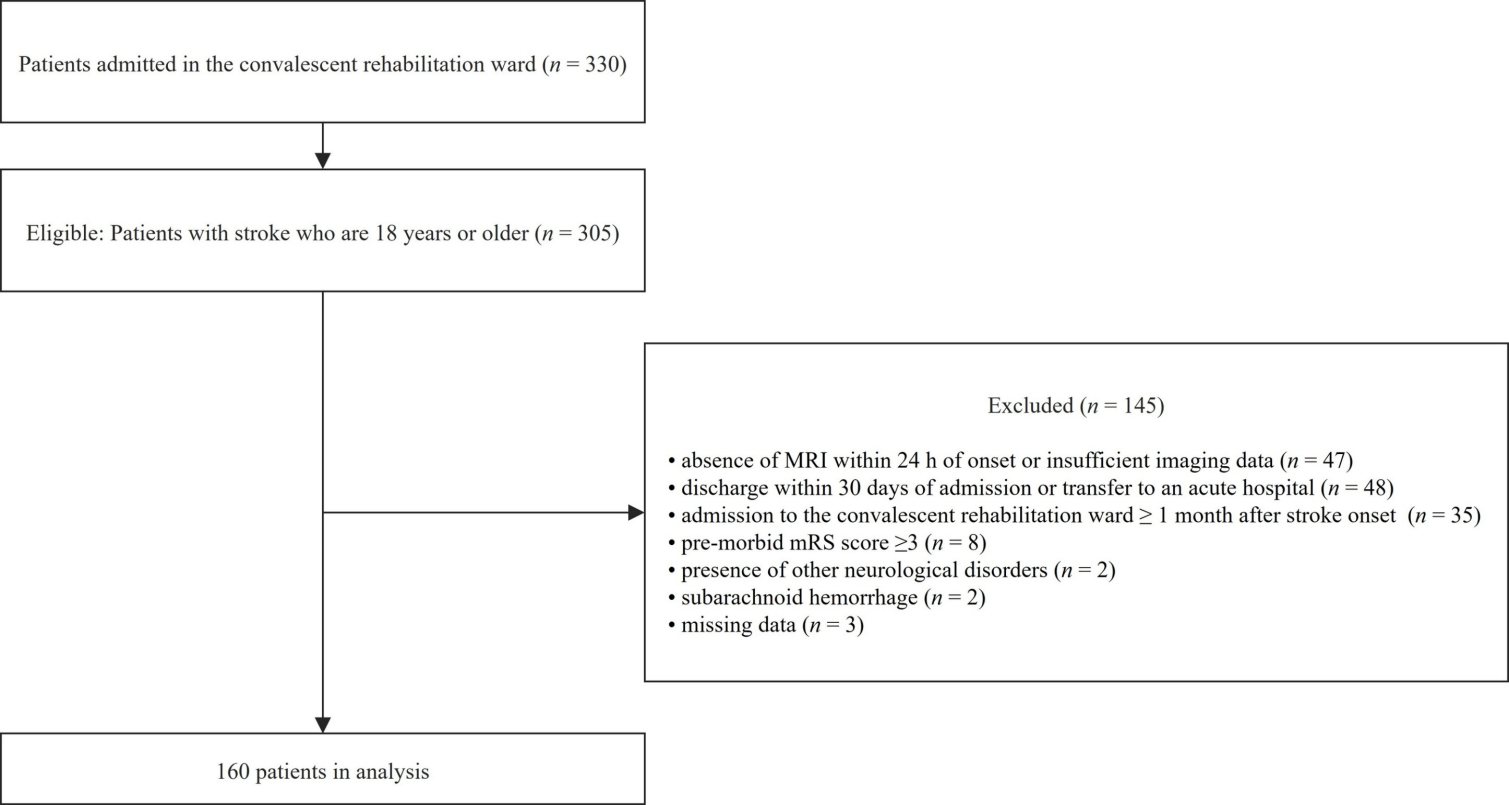
Flow diagram illustrating the inclusion and exclusion criteria.

In total, 305 patients with stroke were admitted to the convalescent rehabilitation ward during the study period. Of these, 145 patients were excluded for the following reasons: absence of MRI within 24 hours of onset or insufficient imaging data (n = 47), admission to the convalescent rehabilitation ward ≥1 month after stroke onset (n = 35), discharge within 30 days of admission to the convalescent rehabilitation ward or transfer to an acute hospital for the treatment of other diseases (n = 48), premorbid mRS ≥3 (n = 8), presence of other neurological diseases (n = 2), subarachnoid hemorrhage (n = 2), and missing data (n = 3). Thus, the final analysis included 160 patients (median age: 73.0 years; IQR: 64.0-80.0 years; male: n = 90, 56.2%). Figure 1 shows a flow diagram of the patient selection process.

The brain frailty scores among the participants were distributed as follows: 14 individuals (8.75%) scored 0, 38 individuals (23.75%) scored 1, 76 individuals (47.50%) scored 2, and 32 individuals (20.0%) scored 3. Brain atrophy was found in 38 individuals (23.8%), WMH were identified in 128 individuals (80.0%), and old vascular lesions were observed in 120 individuals (75.0%).

Table 1 presents the baseline characteristics of the different brain frailty score groups. Significant differences in age (p < 0.001), BMI (p = 0.010), stroke subtype (p = 0.004), and premorbid mRS (p = 0.025) were observed among the four groups. However, no statistically significant differences in FIM-M (p = 0.203) and FIM-C (p = 0.067) scores at the time of admission to the convalescent rehabilitation ward were found among these groups. Furthermore, there were no significant differences in the subtypes of prior stroke, which could potentially influence the brain frailty score, among the four groups (p = 0.090).

**Table 1:** Participants’ characteristics. Values are presented as median (IQR) unless otherwise specified. ^a^Kruskal–Wallis test. ^b^Chi-square test. ^c^One-way analysis of variance. ^d^Fisher’s exact test. ^e^Mean (standard deviation). BMI, body mass index; mRS, modified Rankin scale; CCI, Charlson’s comorbidities index; BRS, Brunnstrom Recovery Stage; FIM-M, Functional Independence Measure motor domain; FIM-C, Functional Independence Measure cognitive domain.

Variables	Total, n = 160	Brain frailty score				p-value
		Score 0, n = 14 (8.75%)	Score 1, n = 38 (23.75%)	Score 2, n = 76 (47.50%)	Score 3, n = 32 (20.00%)	
Age, years	73.0 (64.0–80.0)	61.0 (52.5–70.0)	67.0 (58.0–75.5)	73.0 (65.0–78.0)	82.5 (78.8–87.0)	<0.001^a^
Sex (male), n (%)	90 (56.2%)	12 (85.7%)	17 (44.7%)	44 (57.9%)	17 (53.1%)	0.066^b^
BMI, kg/m^2^	22.5 (20.4–24.9)	23.9 (4.65)^e^	22.7 (4.22)^e^	23.7 (3.66)^e^	21.1 (2.59)^e^	0.010^c^
Stroke subtype, n (%)						0.004^d^
Ischemia	104 (65.0%)	9 (64.3%)	21 (55.3%)	45 (59.2%)	29 (90.6%)	
Hemorrhage	56 (35.0%)	5 (35.7%)	17 (44.7%)	31 (40.8%)	3 (9.4%)	
Days from onset, days	14.0 (11.0–17.3)	15.0 (14.0–17.0)	13.0 (11.0–16.0)	13.5 (11.0–16.3)	15.0 (11.8–20.0)	0.220^a^
Pre-morbid mRS, n (%)						0.025^d^
0	131 (81.9%)	14 (100%)	36 (94.7%)	60 (78.9%)	21 (65.6%)	
1	23 (14.4%)	0 (0.0%)	2 (5.3%)	13 (17.1%)	8 (25.0%)	
2	6 (3.8%)	0 (0.0%)	0 (0.0%)	3 (3.9%)	3 (9.4%)	
CCI, score	0 (0–1)	0 (0–0)	0 (0–1)	0 (0–1)	1 (0–2)	0.138^a^
History of stroke, n (%)	26 (16.2%)	0 (0.0%)	3 (7.9%)	15 (19.7%)	8 (25.0%)	0.064^d^
No history of stroke, n (%)	134 (83.8%)	14 (100%)	35 (92.1%)	61 (80.3%)	24 (75.0%)	0.090^d^
Ischemia, n (%)	20 (12.5%)	0 (0%)	1 (2.6%)	13 (17.1%)	6 (18.8%)	
Hemorrhage, n (%)	6 (3.8%)	0 (0%)	2 (5.3%)	2 (2.6%)	2 (6.2%)	
BRS (lower limb)	5 (3–6)	6 (3.5–6)	5 (3–5)	5 (3–6)	5 (4–5.3)	0.148^a^
Length of stay, days	68.0 (50.0–102.0)	81.0 (45.3–121.8)	88.0 (53.8–124.5)	67.5 (53.0–99.3)	64.5 (43.0–84.5)	0.145^a^
FIM-M at admission, score	47.0 (33.0–67.0)	60.0 (37.5–69.8)	46.0 (32.5–67.8)	54.0 (33.0–68.0)	38.5 (32.0–53.5)	0.203^a^
FIM-C at admission, score	19.0 (14.0–27.0)	16.0 (11.3–23.5)	19.0 (13.3–27.0)	22.0 (16.0–28.0)	17.0 (13.0–21.3)	0.067^a^

**Figure 2:**
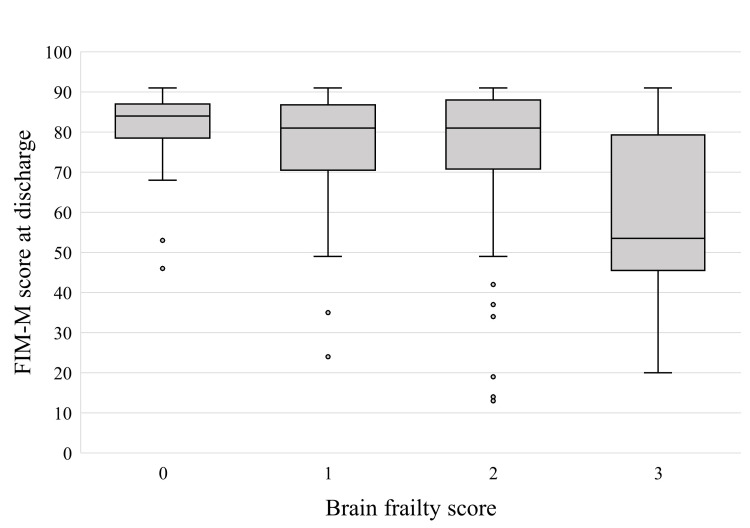
Comparison of FIM-M scores at discharge among the brain frailty score groups.

Figure 2 shows the comparison of FIM-M scores at discharge among the different brain frailty score groups. Participants with a brain frailty score of 3 had significantly lower FIM-M scores at discharge than those with lower brain frailty scores (p = 0.001). 

Table 2 presents the results of the multivariate analysis. All VIF values were below 10, indicating no multicollinearity between the independent variables. The multiple regression analysis revealed that a brain frailty score of 3 was significantly associated with lower FIM-M scores at discharge, even after adjusting for covariates (B = −8.94; 95% CI: −17.52 to −0.37; β = −0.18; p = 0.041).

**Table 2:** Multiple regression analysis for the FIM-M. B, partial regression coefficient; CI, confidence interval; SE, standardized error; β, standardized partial regression coefficient; mRS, modified Rankin scale; CCI, Charlson’s comorbidities index; BRS, Brunnstrom Recovery Stage; FIM-M, Functional Independence Measure motor domain; FIM-C, Functional Independence Measure cognitive domain. Durbin–Watson: 2.08; adjusted R^2^ = 0.66.

Variables	B	95% CI	SE	β	p-value
Age	−0.20	−0.40, 0.01	0.11	−0.12	0.066
Sex (female)	0.32	−3.74, 4.38	2.06	0.008	0.877
Stroke subtype					
Ischemia (reference)	-	-	-	-	-
Hemorrhage	2.78	−1.82, 7.37	2.32	0.07	0.234
Days from onset	−0.67	−1.05, −0.29	0.19	−0.18	0.001
History of stroke	8.22	1.87, 14.57	3.21	0.15	0.012
Premorbid mRS					
0 (reference)	-	-	-	-	-
1	−3.49	−10.11, 3.13	3.35	−0.06	0.299
2	−7.70	−17.99, 2.59	5.21	−0.07	0.141
CCI	−2.80	−4.65, −0.95	0.94	−0.17	0.003
BRS (lower limb)	3.01	0.94, 5.08	1.05	0.21	0.005
FIM-M at admission	0.43	0.23, 0.62	0.10	0.44	<0.001
FIM-C at admission	0.37	−0.05, 0.78	0.21	0.14	0.085
Brain frailty score					
0 (reference)	-	-	-	-	-
1	−1.69	−9.21, 5.84	3.81	−0.04	0.658
2	−3.83	−11.12, 3.46	3.69	−0.10	0.300
3	−8.94	−17.52, −0.37	4.34	−0.18	0.041

**Figure 3:**
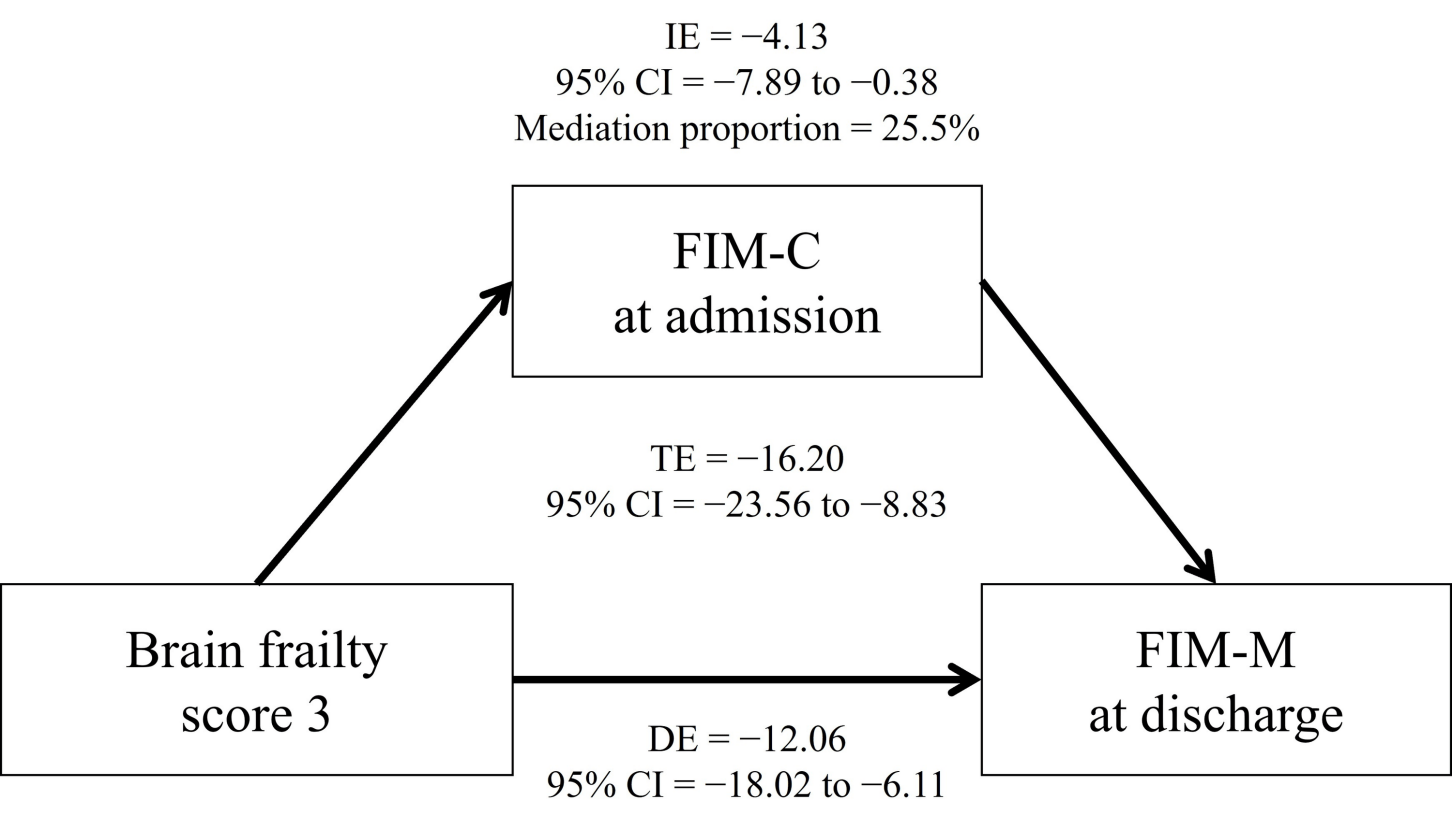
Severe brain frailty is directly and indirectly associated with FIM-M score at discharge (TE = −16.20; DE = −12.06; IE = −4.13). FIM-C, Functional Independence Measure cognitive domain; FIM-M, Functional Independence Measure motor domain; IE, indirect effect; DE, direct effect; TE, total effect; CI, confidence interval.

Figure 3 presents the results of the mediation analysis. The mediation analysis revealed that a severe brain frailty score (score 3) had a significant total effect on the FIM-M score at discharge (total effect: −16.20; 95% CI: −23.56 to −8.83; p < 0.001). Furthermore, a brain frailty score of 3 exhibited a significant direct effect on the FIM-M score (direct effect: −12.06; 95% CI: −18.02 to −6.11) and a significant indirect effect mediated by the FIM-C score at admission (indirect effect: −4.13; 95% CI: −7.89 to −0.38; mediation proportion: 25.5%). In contrast, the other brain frailty scores did not significantly affect the FIM-M score (score 1: p = 0.235; score 2: p = 0.155).

## Discussion

This study examined the relationship between brain frailty scores, assessed using cranial MRI at stroke onset, and physical function at discharge in patients with stroke admitted to a convalescent rehabilitation ward. The key findings of this study were as follows: (1) the prevalence of brain frailty and its components in patients with stroke and (2) a negative association between severe brain frailty scores and ADLs at discharge from the convalescent rehabilitation ward.

In this study, the prevalence of brain frailty was observed as follows: 14 individuals (8.75%) with a score of 0, 38 individuals (23.75%) with a score of 1, 76 individuals (47.50%) with a score of 2, and 32 individuals (20.0%) with a score of 3, suggesting that more than half of the participants had moderate to severe brain frailty. Among the imaging markers constituting brain frailty, WMH (n = 128, 80.0%) and old vascular lesions (n = 120, 75.0%) were the most prevalent, followed by brain atrophy (n = 38, 23.8%). Previous studies have reported an association between brain frailty and age [13]. This study targeted patients in Japan, which has a high rate of aging. This demographic factor may explain the higher prevalence of brain frailty observed in our findings. In previous research, brain frailty and cerebral small vessel disease (CSVD) (e.g., lacunes, cerebral microbleeds, and enlarged perivascular spaces) were found to be associated with stroke events [13,27], mortality [12,27,28], and adverse outcomes [9,11,14,27-29]. Considering these findings alongside our results, it can be suggested that brain frailty is not an uncommon condition in patients with stroke and may be an important imaging marker that warrants routine evaluation in clinical practice.

A higher burden of brain frailty was negatively associated with ADLs at discharge. Furthermore, cognitive function partially mediated the relationship between brain frailty and ADLs. This study is the first to demonstrate the relationship between brain frailty and physical function at discharge among patients with stroke admitted to a convalescent rehabilitation ward. In this study, only severe brain frailty was found to be associated with ADLs at discharge, while no significant associations were observed for other brain frailty scores. This finding suggests a potential threshold effect, wherein the presence of all three components constituting brain frailty may have exerted a negative impact on physical function. The components of brain frailty, such as WMH, old vascular lesions, and brain atrophy, are associated with cognitive and functional outcomes in older adults and patients with stroke [9,30-37]. In particular, WMH has been linked to walking and balance abilities [32-35] and the presence and severity of urinary incontinence [38], all of which are key elements of ADLs. These factors are reflected in the self-care and mobility items of the FIM-M score, potentially explaining the observed negative association with FIM-M scores at discharge.

Furthermore, cognitive function may also contribute to the relationship between brain frailty and ADLs. Brain frailty and its components are associated with cognitive impairment and dementia in both older adults and patients with stroke [9,30,31]. Similarly, CSVD has been implicated in brain network alterations, which are associated with cognitive function [39] and cognitive impairment [36]. A recent systematic review also reported that cognitive decline is associated with reduced ADLs, IADLs, and participation restrictions [40]. Thus, brain frailty may have contributed to lower ADLs at discharge. As cognitive function partially mediates the relationship between brain frailty and physical function, stroke patients with severe brain frailty may benefit from, in addition to training aimed at enhancing physical function, cognitive training designed to improve memory and attention, which could contribute to the improvement of ADLs.

Strengths and limitations of the study

Cranial MRI is frequently performed as part of routine clinical practice in the early phase after stroke onset. This study highlights that brain frailty can be assessed using routinely acquired MRI data without requiring additional evaluations, making it a practical and accessible tool for clinical application. The findings suggest that severe brain frailty serves as a significant prognostic indicator of functional outcomes in patients undergoing rehabilitation. The brain frailty score demonstrates high interrater reliability (ICC = 0.93) and can be easily assessed even by nonphysician healthcare professionals. Therefore, incorporating brain frailty assessment into routine clinical practice may facilitate the early identification of patients at higher risk for poor recovery, enabling the development of more personalized and targeted rehabilitation strategies, such as cognitive and ADL training.

This study has several limitations. First, as it was conducted in Japan, a country with a highly aged population, the age distribution may differ from that of other countries. Additionally, since this was a single-institution study with a small sample size, these factors may limit the generalizability of the findings. Furthermore, in patients with intracerebral hemorrhage, CT is more commonly performed than MRI, which may also affect the applicability of the results. Second, because this was a retrospective study, unmeasured confounding factors, such as the National Institutes of Health Stroke Scale score at onset and the effects of rehabilitation, may be present. Given that stroke severity at onset and rehabilitation interventions can influence physical function at discharge, future studies should incorporate these confounders to further elucidate the relationship between brain frailty and physical function. Third, because this study utilized both 1.5-Tesla and 3-Tesla MRI scanners, variability in image resolution may have influenced the assessment of brain frailty. Future research should conduct sensitivity analyses to evaluate the impact of MRI settings on the robustness of the findings. Further research is necessary to explore the association between brain frailty and other outcomes beyond ADLs and long-term outcomes following discharge.

## Conclusions

This study investigated the relationship between brain frailty and physical function among patients with stroke undergoing rehabilitation. The findings indicate that moderate to severe brain frailty was observed in nearly half of the patients, suggesting that brain frailty is not uncommon in this population. Furthermore, a higher burden of brain frailty was negatively associated with ADLs at discharge. These findings underscore the potential importance of incorporating brain frailty assessment into routine clinical practice, as early identification of brain frailty could help predict rehabilitation outcomes and guide individualized rehabilitation strategies. Future research should conduct multicenter prospective studies with larger sample sizes, incorporating unmeasured confounding factors to further elucidate the relationship between brain frailty and physical function.
